# Investigation of Fatigue Behavior of ABS and PC-ABS Polymers at Different Temperatures

**DOI:** 10.3390/ma11101818

**Published:** 2018-09-25

**Authors:** Andrea Mura, Alessando Ricci, Giancarlo Canavese

**Affiliations:** 1 Department of Mechanical and Aerospace Engineering, Politecnico di Torino, C.so Duca degli Abruzzi 24, 10129 Torino, Italy; 2 OLSA S.p.a, Corso Allamano 70, 10098 Rivoli (TO), Italy; alessandro.ricci@olsagroup.com; 3 Department of Applied Science and Technology, Politecnico di Torino, C.so Duca degli Abruzzi 24, 10129 Torino, Italy; giancarlo.canavese@polito.it

**Keywords:** fatigue, temperature, plastic, polymers, ABS, PC-ABS

## Abstract

Plastics are widely used in structural components where cyclic loads may cause fatigue failure. In particular, in some applications such as in vehicles, the working temperature may change and therefore the strength of the polymeric materials. In this work, the fatigue behavior of two thermoplastic materials (ABS and PC-ABS) at different temperatures has been investigated. In particular, three temperatures have been considered representing the working condition at room temperature, at low temperature (winter conditions), and high temperature (summer conditions and/or components close to the engine). Results show that high temperature have big impact on fatigue performance, while low temperatures may also have a slight positive effect.

## 1. Introduction

Polymeric materials are widely used in many industrial fields to produce a vast range of components, form the ones with mainly decorative functions, to others that allow also structural and working functions [1]. In particular, in automotive applications plastic materials are subjected to mechanical and thermal loads. Mechanical loads are mainly due to structural functions or vibrations, these solicitations are variable with the time and may cause fatigue failures [2,3]. Moreover, components installed on vehicles work at temperatures that may vary according to both working and/or environmental conditions. As a matter of fact, the mechanical strength of plastic material strongly depends on the temperature [4].

Therefore, it is important to characterize plastic materials used in this field knowing their fatigue behavior and the corresponding fatigue limits at different temperatures, in order to give the designers the right pieces of information to properly design components.

In the literature, only few works are available about fatigue behavior of plastic materials, and even less about the fatigue characterization at temperatures different form the room one. Most works have been done in the last decades, investigating different aspects of fatigue in polymers. The fracture micromechanisms and crack propagation of plastic materials has been investigated by Hertzberg et al. [5], proposing a model to characterize crack growth [6]. Fatigue crack growth has been investigated by Radon [7] in both uniaxial and 2D stress. Beardmore [8] worked on cycled response analyzing phenomena due to hysteresis. Hertzberg et al. [9], Arad et al. [10], and Wyzgoski et al. [11] focused on the effect of frequency on fatigue crack growth, finding that its effect is affect crack propagation and fatigue life. Other authors—such as Prevorsek et al. [12], Andrews [13], McEvily et al. [14], and Lake et al. [15]—investigated the crack nucleation and propagation, finding some common aspect between the fatigue mechanisms of polymers and metals [16,17]. Spandan et al. [18] proposed a cohesive model to simulate fatigue crack propagation in polymeric materials. Ramsteiner et al. [19] considered also the influence of the shape of the specimen on crack propagation. Klimkeit et al. [20] investigated the multiaxial fatigue behavior reinforced polymers.

More recently, Ravi Chandran [21] proposed a theory for the mechanical fatigue behavior of polymers, in the absence of thermal effects. Ding et al. [22] evaluated the crack growth with a numerical approach based on plastically dissipated energy. Amir et al. [23] and Jones et al. [24] investigated the self-heating effects on fatigue life.

This work focuses on aspects that are still not fully investigated in the literature, in particular the effect of high and low temperature of fatigue life of ABS and PC-ABS polymers.

The investigation has been carried on by means of experimental tests performed on samples, obtained by moulding injection, at room temperature, high temperature (85 °C), and low temperature (−27 °C).

## 2. Experimental Set-Up

### Materials and Methods

In this work, two polymeric materials have been investigated: ABS (Acrylonitrile butadiene styrene) commercialized as SicoflexS460 and produced by Ravago Group company (Luxembourg City, Luxembourg), with density 1.04 g/cm^3^ and PC-ABS (a blend of polycarbonate (PC) and acrylonitrile butadiene styrene), commercialized as Cycoloy1200HF and produced by Sabic company (Riad, Arabia Saudita), with density 1.15 g/cm^3^.

The glass transition temperature Tg of ABS is about 96 °C and for PC-ABS is about 125 °C.

Specimens have been made by means of mold injection.

The fatigue limits have been obtained using six samples, according to the Dixon method [25].

Samples dimensions and geometry have been made according to specimen type 1A ISO 527-2 standard.

Tensile tests have been done before to proceed with fatigue tests by means of a MTS QTEST Elite (Eden Prairie, MN USA) electromechanical testing machine.

**Figure 1:**
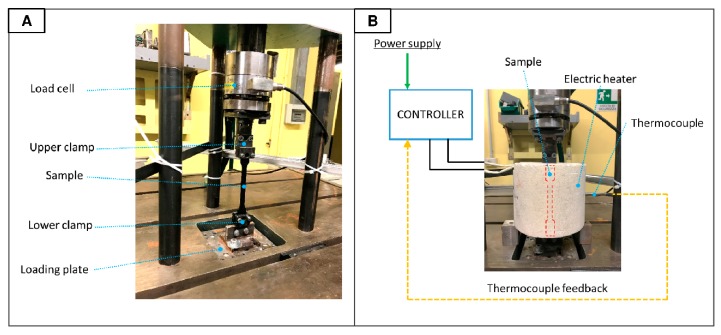
Experimental set-up for room temperature tests (**A**) and high temperature tests (**B**).

Fatigue tests have been performed in tension by means of a mechanical Baldwin fatigue tester SF-1-U. The machine working frequency is 30 Hz, the sample clamping is the machine is represented in Figure 1A.

Both fatigue and tensile tests have been performed at room temperature (22 °C), 85 °C, and −27 °C, the average relative humidity during the tests was 68%. Concerning tests at room temperature, the temperature of the specimens has been measured during the tests in order to check if the test frequency could increase the material temperature and so on influence the results. The increase of specimen temperature was less than 1 °C and therefore negligible.

Tests at high temperature (85 °C) have been performed using a cylindrical electric heater (WATLOW 0624—650 W) controlled in closed loop by a thermocouple and a PID controller (WATLOW EC) as represented in Figure 1A. The temperature variation during the tests was ± 3 °C.

**Figure 2:**
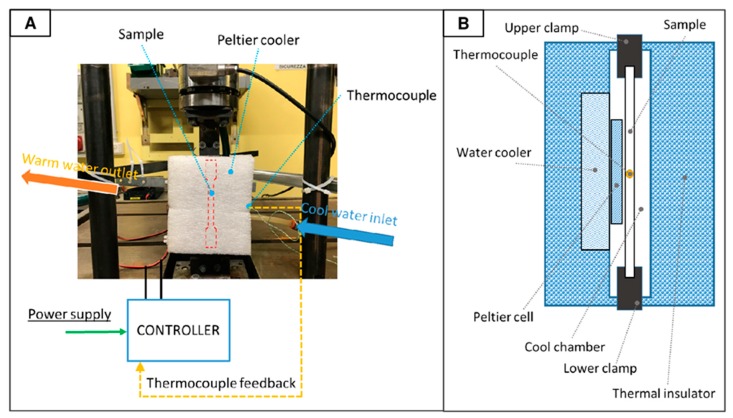
Experimental set-up for low temperature tests: equipment mounted in the fatigue machine (**A**) and schematic detail of the water–Peltier cooler (**B**).

Tests at low temperature (−27 °C) have been performed by means of a dedicated chiller based on liquid cooled Peltier cell, controlled by a dedicated driver. The picture and schematic of the chiller are represented respectively in Figure 2A,B. The specimen temperature has been monitored during the tests by a thermocouple; the temperature variation during the tests was ± 0.1 °C. Before starting the tests at high and low temperatures, the specimen temperature was stabilized for 10 min.

## 3. Results and Discussion

### 3.1. Tensile Behavior

**Figure 3:**
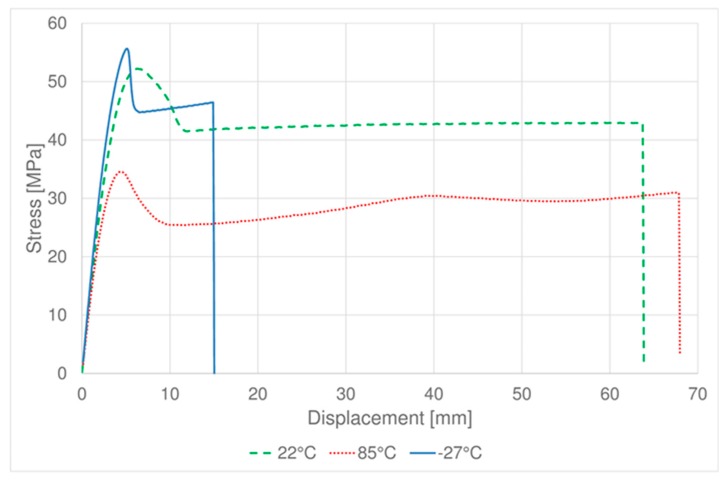
Tensile test of PC-ABS: average results at different temperatures.

**Figure 4:**
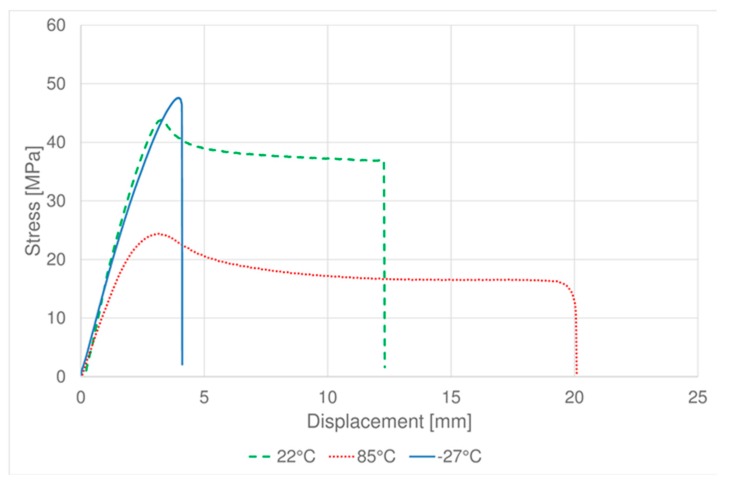
Tensile test of ABS: average results at different temperatures.

Figure 3 and Figure 4 show the tensile tests average results obtained respectively for PC-ABS and ABS samples. It is possible to observe that for PC-ABS at the three testing temperatures, the behavior is typical as for the ductile materials, while at −27 °C ABS the behavior became brittle [26]. The total elongation is strongly affected by the temperature, as expected, at low temperature the elongation to break is lower than at high temperatures.

Table 1 resumes the tensile results obtained for the two analyzed materials at different temperatures, along with the standard deviation. Tensile stresses at break and yield stresses have been obtained according to ISO 527-1 standard [26].

**Figure 5:**
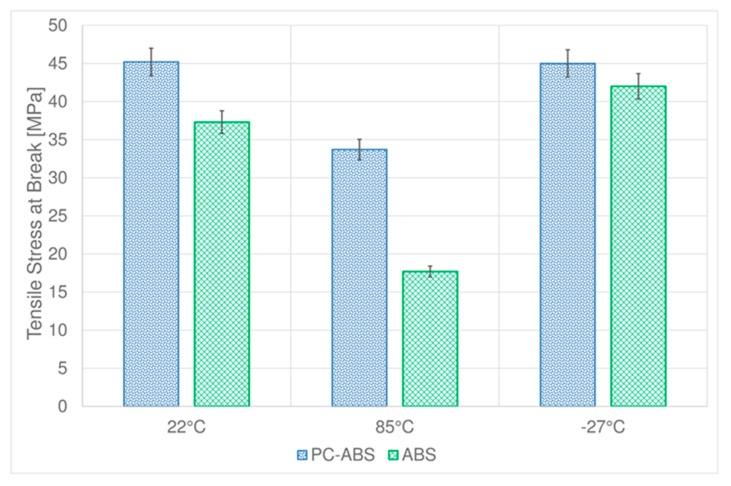
Comparison between tensile stress at break of PC-ABS and ABS at different temperatures.

The tensile stress at break values (Figure 5) obtained for PC-ABS at room temperature and low temperature are nearly the same (the value at −27 °C is slightly lower respect to room temperature), while it reduces of about 25% at high temperature. Concerning ABS samples, the tensile stress at break at low temperature is higher respect to the one obtained at room temperature, but it has to be considered that in this case, as shown before, this material changes its behavior from ductile to brittle. At 85 °C, tensile stress at break of ABS is less than half compared to room temperature.

**Figure 6:**
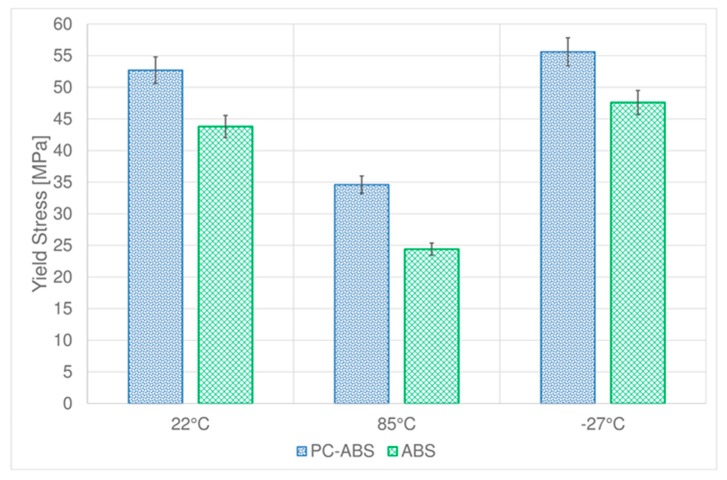
Comparison between yield stresses of PC-ABS and ABS at different temperatures.

Yielding stress of PC-ABS (Figure 6) increases of about 5% at −27 °C respect to the value obtained at room temperature, but it decreases of almost 35% at 85 °C.

The behavior of yielding stress in ABS (Figure 6) is similar to the one obtained for tensile stress at break, it increases at low temperature and deeply decreases at 85 °C.

### 3.2. Fatigue Behavior

The fatigue limits obtained by Dixon method [25] for the considered materials are resumed in Table 2, along with the standard deviation.

**Figure 7:**
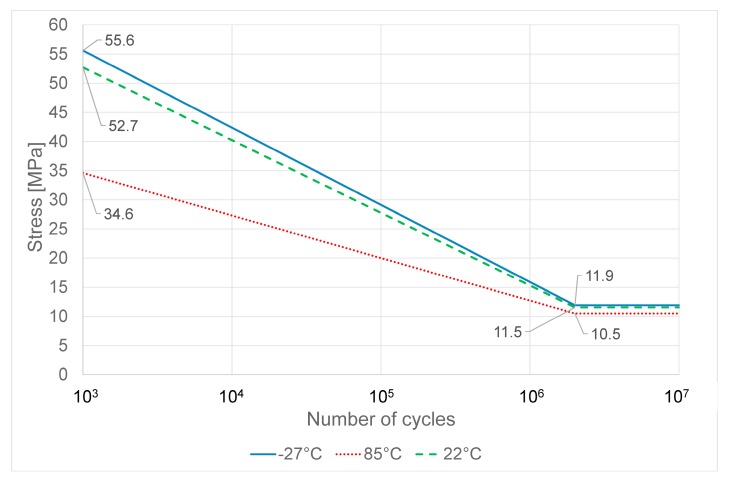
Wöhler curve at 50% survival for PC-ABS at different temperatures.

**Figure 8:**
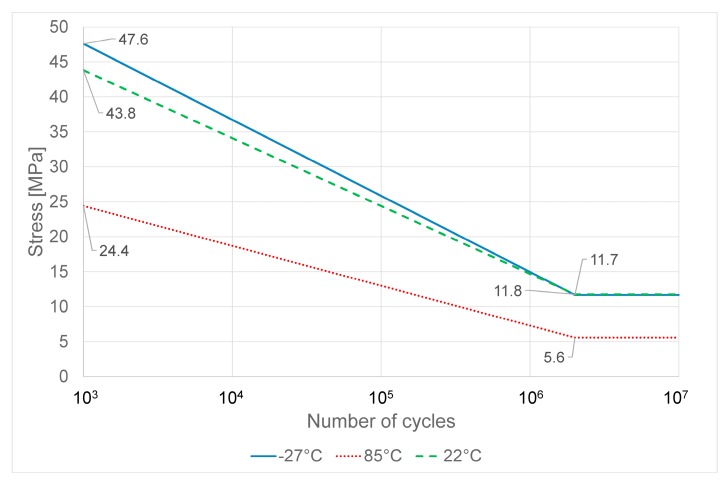
Wöhler curve at 50% survival for ABS at different temperatures.

The simplified Wöhler curves obtained for PC-ABS and ABS at different temperatures are shown respectively in Figure 7 and Figure 8. The simplified Wöhler curves have been obtained by the average tensile parameters shown in the previous section and the fatigue limits shown in Table 1.

Generally speaking, the fatigue limit of both the two examined materials is strongly affected by high temperature. In particular, high temperature reduces the fatigue limit.

**Figure 9:**
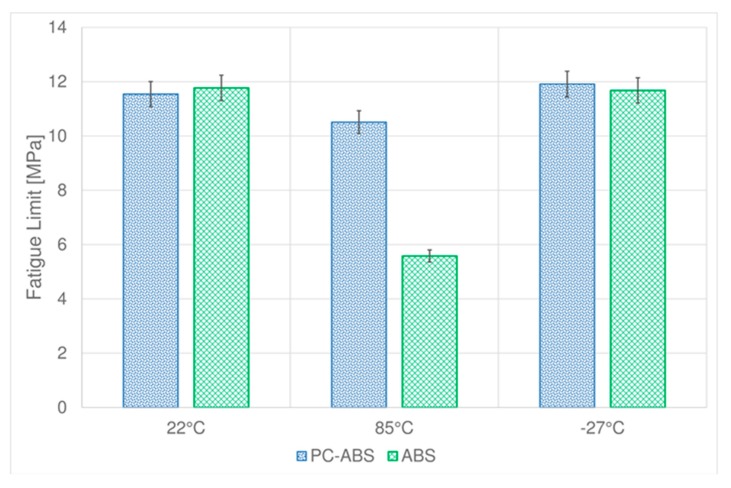
Comparison between fatigue limit of PC-ABS and ABS at different temperatures.

By comparing the performance of PC-ABS and ABS (Figure 9), it is possible to observe that at room temperature the two materials have almost the same fatigue limit. At low temperature, the fatigue limit is slightly higher for PC-ABS and nearly unchanged for ABS.

As fatigue behavior of thermoplastic material is mainly driven by thermal softening and fatigue cracking [3,27,28,29] the slight improvement of fatigue strength at low temperature for PC-ABS could be explained as the increase of temperature due to cyclic stress [29] is reduced by the test’s low temperature bringing lower material damage.

The main differences are found at high temperature where both materials reduce their fatigue limit, but ABS has the worst performance, showing almost half of the fatigue limit of PC-ABS. The reduction of fatigue performance at high temperature is justified as the creep phenomena in thermoplastic polymers increase dramatically with the temperature [30]. The better performance of PC-ABS at high temperature is due to the polycarbonate in the blend that improve mechanical strength of the material, including creep behavior [30].

**Figure 10:**
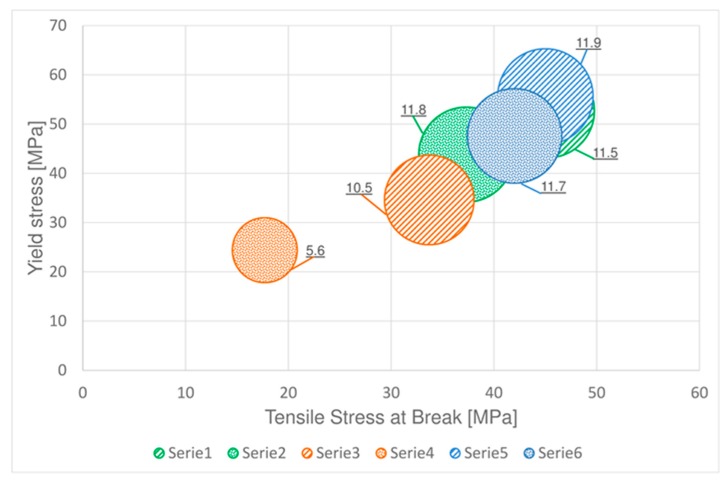
Comparison of tensile and fatigue strength at different temperatures, the circle diameters represent the fatigue limit in MPa.

Figure 10 resumes in one plot the global performance of the two analyzed materials in terms of tensile and fatigue strength and the influence of the temperature. In particular, the horizontal axis represents the tensile stress at break, the vertical axis the yield stress therefore, the center of each circle is given by the tensile properties at a given temperature and the diameter of the circles represent the fatigue limits. This representation highlights that the performance of ABS is heavily reduced at high temperature in the other hand the strength of both PC-ABS and is slightly improved at low temperature.

The fracture surfaces have been analyzed for both the materials by optical (Leica DM4000M, Leica Microsystems, Wetzlar, Germany) and field-emission scanning electron microscopy (FESEM, Zeiss Supra 40, Zeiss, Oberkochen Germany). For the FESEM analysis the samples were previously metalized with a thin film (around 10 nm) of platinum (Pt) by the sputtering system Q150TES (Quorum Technology) operating at 50 mA for 40 s at room temperature and 8 × 10^−4^ mbar.

**Figure 11:**
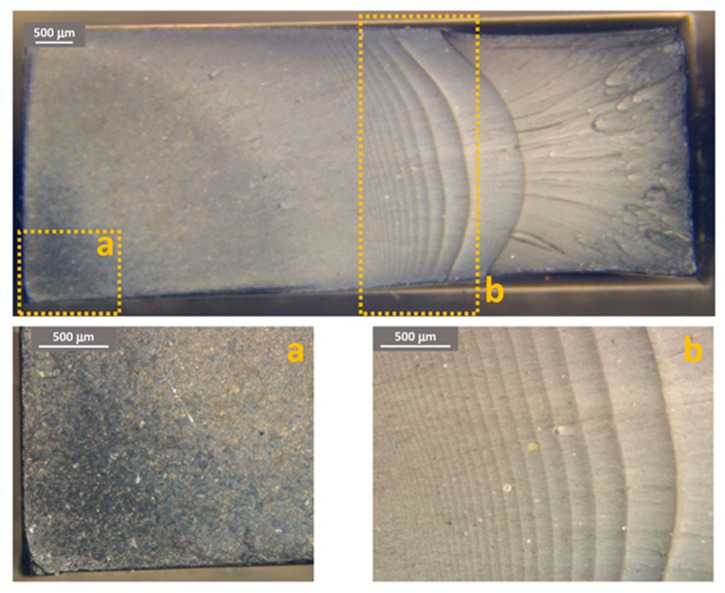
PC-ABS fracture surface after test at room temperature. (**a**) Crack initiation point; (**b**) propagation beach marks.

**Figure 12:**
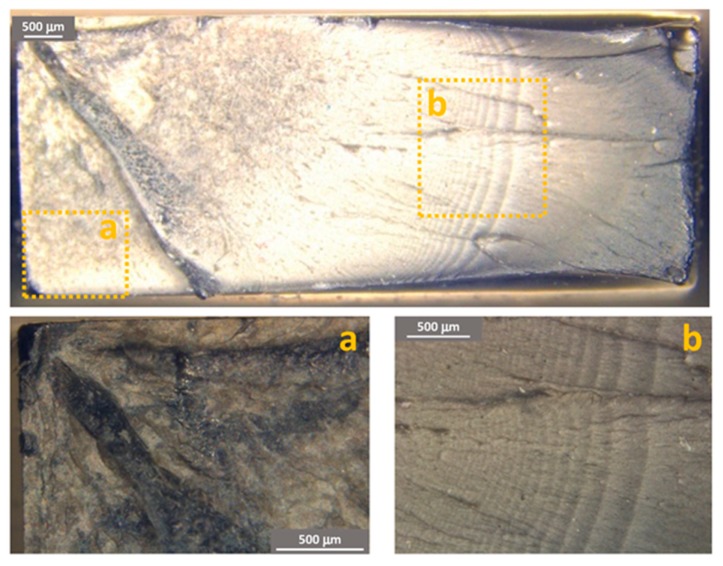
PC-ABS fracture surface after test at 85 °C. (**a**) Crack initiation point; (**b**) propagation beach marks.

**Figure 13:**
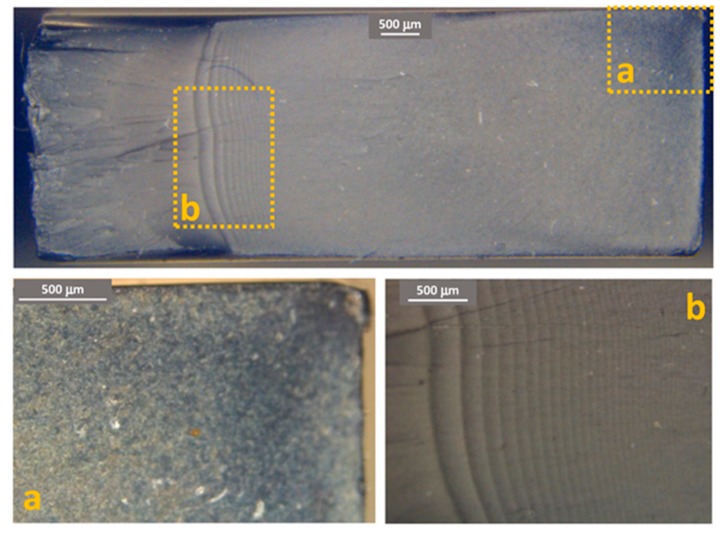
PC-ABS fracture surface after test at −27 °C. (**a**) Crack initiation point; (**b**) propagation beach marks.

**Figure 14:**
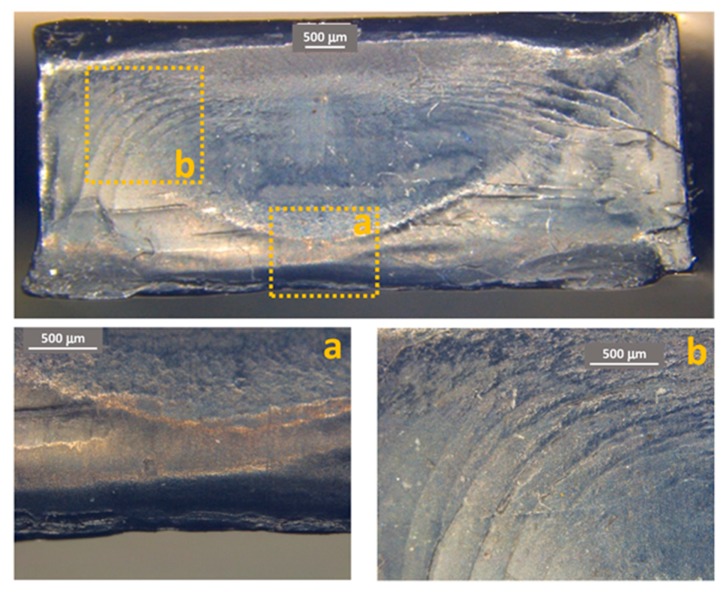
ABS fracture surface after test at room temperature. (**a**) Crack initiation point; (**b**) propagation beach marks.

**Figure 15:**
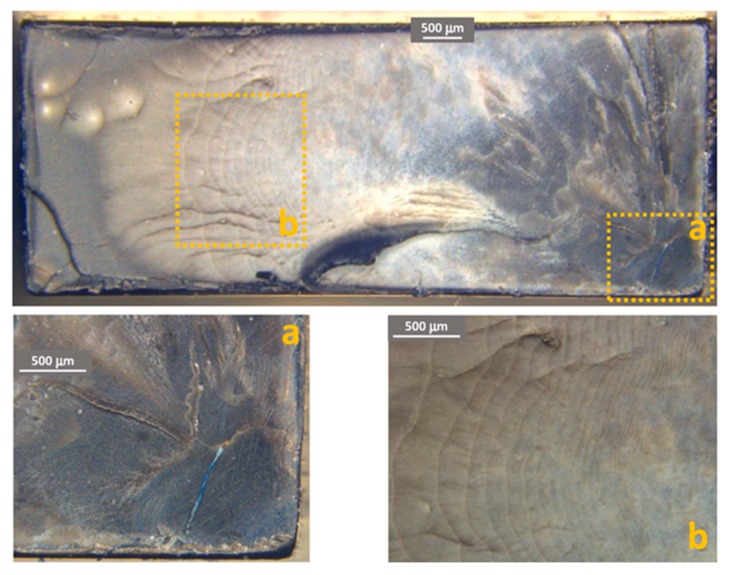
ABS fracture surface after test at 85 °C. (**a**) Crack initiation point; (**b**) propagation beach marks.

**Figure 16:**
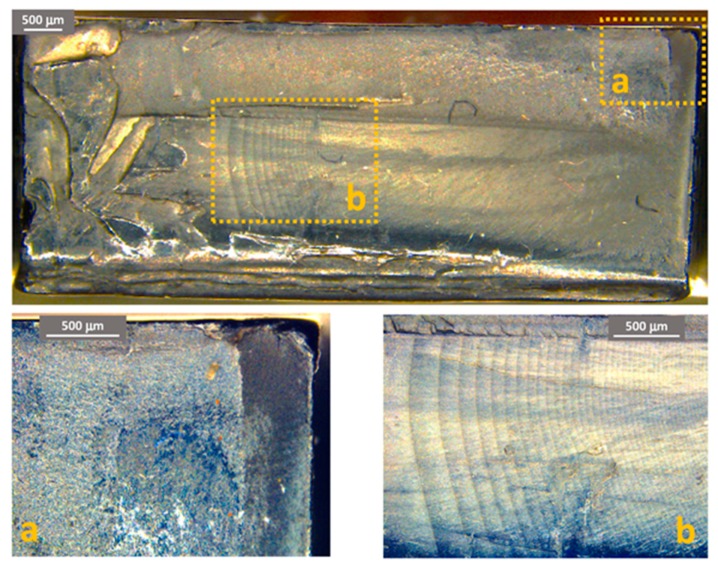
ABS fracture surface after test at −27 °C. (**a**) Crack initiation point; (**b**) propagation beach marks.

Figure 11, Figure 12 and Figure 13 show some examples of fracture surfaces captured by optical microscope of PC-ABS after tests performed at respectively room temperature, 85 °C and −27 °C. Fracture surfaces at different temperatures of ABS, obtained by optical microscope, are shown in Figure 14, Figure 15 and Figure 16. In all the images, the crack initiation point and the discontinuous growth bands are highlighted.

The aspect of the fracture surface of both materials, after testing at room temperature and low temperature is similar, and the crack propagation surface is well defined and slightly rough. In testing at high temperature, the crack propagation surface is smoother; this could be due to the material softening.

Discontinuous growth bands have been found in all the broken samples and their aspect is similar to those found in other polymers after fatigue tests [2,3,27], showing that the material failure is due to fatigue cracking at all the tested temperatures, as fatigue melt fractures are not present even in tests performed at 85 °C.

**Figure 17:**
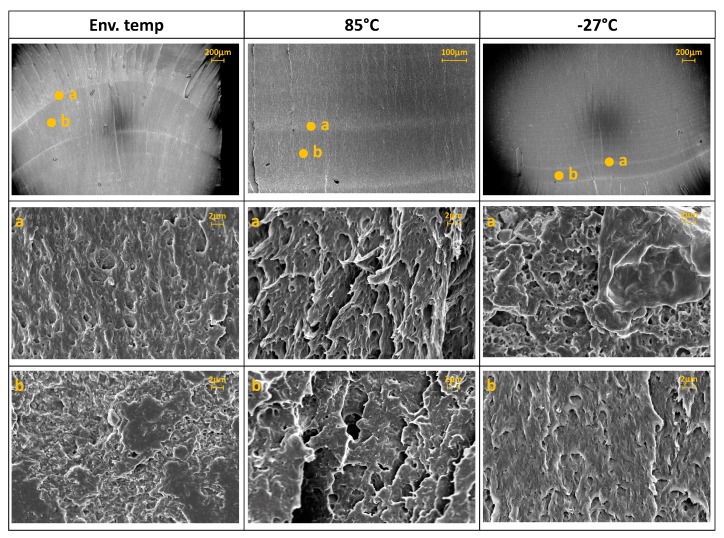
FESEM images of PC-ABS growth bands at different test temperature and magnification of the arrest band (**a**) and between bands (**b**).

**Figure 18:**
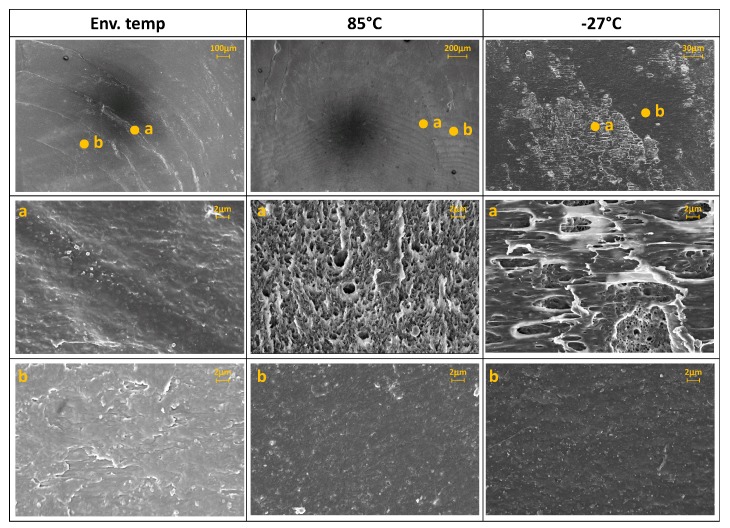
FESEM images of ABS growth bands at different test temperature and magnification of the arrest band (**a**) and between bands (**b**).

Figure 17 and Figure 18 show the FESEM images of the growth band region respectively PC-ABS and ABS samples at different testing temperatures. In the two images, magnifications of the arrest band and of the zone between two arrest bands are identified by the letters (a) and (b). From the images, it is possible to observe that the surface texture on the arrest band is rougher respect to the propagation zone for both the materials and for all the test temperatures. In particular, the area within arrest bands is characterized by many indentations due to the break mechanism. In ABS samples, the propagation zone is smoother with respect to PC-ABS samples, because of the hammering effect during crack propagation.

## 4. Conclusions

In this work, the tensile and fatigue performance of two polymers (PC-ABS and ABS) at different temperatures have been investigated. In particular tests have been performed at room temperature, high temperature (85 °C) and low temperature (−27 °C).

Considering tensile properties, PC-ABS shows a slight improvement yielding stress at low temperature while no influence is shown about tensile stress at break. ABS shows an improvement of both tensile stress at break and yielding stress at low temperature, but it must be highlighted that this material changes its behavior form ductile to brittle at low temperature. Both materials reduce their tensile properties at high temperature, but the performance of ABS are worst respect to PC-ABS.

Concerning the fatigue behavior, the fatigue limit of PC-ABS and ABS is slightly improved at low temperature. At high temperature, PC-ABS reduces its fatigue limit of about 10% while ABS shows a deep reduction of more than half respect to the fatigue limit at room temperature.

The strength variations with the temperature shown by the analyzed materials should be taken into account by designers when designing components that works with not constant temperature, in order to avoid uneven failures and to better predict the components life.

In conclusion, both materials show a good tensile and fatigue behavior at low temperature, at high temperature PC-ABS shows better performance respect to ABS, especially concerning the fatigue limit.

## Figures and Tables

**Table 1:** Tensile parameters of PC-ABS and ABS at different temperatures

	PC-ABS				ABS			
Test Temperature (°C)	Stress at Break (MPa)	Standard Deviation	Yield Stress (MPa)	Standard Deviation	Stress at Break (MPa)	Standard Deviation	Yield Stress (MPa)	Standard Deviation
**22**	45.2	1.2	52.7	0.6	37.3	0.6	43.8	1.2
**85**	33.7	1.7	34.6	1.2	17.7	1.5	24.4	1.3
**−27**	45.0	0.9	55.6	0.7	42.0	0.2	47.6	0.5

**Table 2:** Fatigue limits of PC-ABS and ABS at different temperatures

	PC-ABS		ABS	
Test Temperature (°C)	Fatigue Limit (MPa)	Standard Deviation	Fatigue Limit (MPa)	Standard Deviation
22	45.2	0.6	37.3	0.7
85	33.7	0.4	17.7	0.3
−27	45.0	0.8	42.0	0.7

## Data Availability

The raw data required to reproduce these findings cannot be shared at this time due to time limitations.
